# Quantifying energy transition vulnerability helps more just and inclusive decarbonization

**DOI:** 10.1093/pnasnexus/pgae427

**Published:** 2024-10-22

**Authors:** Yifan Shen, Xunpeng Shi, Zhibo Zhao, R Quentin Grafton, Jian Yu, Yuli Shan

**Affiliations:** Department of Economics and Finance, SILC Business School, Shanghai University, Shanghai 201899, China; Australia-China Relations Institute, University of Technology Sydney, Sydney, NSW 2007, Australia; Department of Finance, School of Finance, Qilu University of Technology (Shandong Academy of Sciences), Jinan 250100, China; Crawford School of Public Policy, The Australian National University, Acton, ACT 2601, Australia; School of Economics, Central University of Finance and Economics, Beijing 102206, China; School of Geography, Earth and Environmental Sciences, University of Birmingham, Birmingham B15 2TT, United Kingdom

## Abstract

The COP28 agreement signals “beginning of the end” of the fossil fuel era, calling on countries to contribute to global efforts to transition away from fossil fuels in energy systems in a just, orderly and equitable manner. While a quantitative assessment of country's vulnerability in energy transition is a prerequisite for national and international policy makers to ensure a just and inclusive transition, it is notably absent in the existing research. Here, we develop a conceptual framework based on the vulnerability scoping diagram (VSD) method to assess differences in energy transition vulnerability across countries, with a specific focus on the challenges associated with transitioning away from fossil fuels. The resulting energy transition vulnerability index (ETVI) scores reveal that countries in the Global South generally exhibit higher vulnerability in their energy transition compared to those in the Global North, and this gap has widened over the past decade. Moreover, the COVID-19 pandemic has disrupted the decade-long trend of continuous decline in global energy transition vulnerability. This study also provides two important applications of ETVI scores, aligning them with major global sustainable development agenda. Firstly, we identify substantial differences in the dynamics of transition vulnerability across seven major party groups in the international climate change negotiations and distinguish four energy transition statuses in relation to achieving global climate goals: *Stressful*, *Leapfrog*, *Potential Challenges*, and *Less Painful*. Secondly, we demonstrate crucial synergies between energy transition resilience and the 2030 United Nations Sustainable Development Goals (SDGs).

**Significance Statement**

The COP28 agreement marks a pivotal moment, signifying the “beginning of the end” of the fossil fuel era and calling upon nations to transition away from fossil fuels in their energy systems in a just, orderly and equitable manner. In this study, we support the global just transition efforts by developing a novel measure of energy transition vulnerability across countries. Our findings show that countries in the Global South generally exhibit higher vulnerability in their energy transition compared to those in the Global North, and this gap has widened over the past decade. We also provide two important applications, demonstrating how our measure helps to achieve major global sustainable development agendas, such as the Paris climate goals and the SDGs.

## Introduction

The transition away from fossil-based toward low-carbon energy systems is critical to address the impacts of climate change and ensure a sustainable future since the energy sector accounts for two-thirds of global greenhouse gas emissions (1, 2). This energy transition also needs to respond to the economic, social, and environmental impacts on historically marginalized and vulnerable stakeholders. Moreover, a successful energy transition must contend with price shocks, energy supply disruptions, and socioeconomic hardships that are unlikely to be evenly distributed across individuals, communities, and nations (3). For example, the accompanying hardships of the energy transition could be disproportionately greater in regions or countries with more exposure to fossil fuels, greater sensitivity to energy price changes, and which have limited financial or technological capability to attenuate, cope, or mitigate the potential negative impacts. Thus, it is of critical importance to ensure that the global energy transition does not cause to an unfair distribution of benefits and costs, noting that distributional justice is a key tenet of energy justice (3–5).

Identifying energy transition vulnerability is a precondition for implementing an economically, socially, and environmentally just energy transition thus supports energy justice. Here, energy justice begins with questioning how the benefits and losses are distributed, and who is most affected (5). Assessment of vulnerability—defined as “the propensity or predisposition to be adversely affected and encompasses a variety of concepts and elements, including sensitivity or susceptibility to harm and lack of capacity to cope and adapt” (6)—is a key tool to identify vulnerable stakeholders, such as nations, communities, and players. While there have been sustained efforts to delineate the vulnerability framework, especially in relation to climate change (7–9), more is required. In particular, given the urgent need to achieve immediate and more ambitious transitions to limit global warming to less than 2 °C (10), measures of energy transition vulnerability provide important guidance about the preferred and sustainable decarbonization pathways.

Despite the growing emphasis on achieving a just energy transition across regions, as evidenced by works like Carley et al. (11) and Shi et al. (12), there remains a notable gap in conducting comprehensive assessments of energy transition vulnerabilities on a global scale. Such assessments are crucial for informing national policy-making and facilitating international collaboration on climate initiatives. While recent studies on energy transition vulnerability span various scales, encompassing countries (13), cities (14), communities (15), households (16), and power generation sectors (17), many rely predominantly on qualitative methods like questionnaires, interviews, and focus group discussions.

While qualitative research provides valuable and nuanced insights resilient to uncertainties, there is also a need for quantitative approaches to enhance policy-making and to monitor the progress of transition vulnerability across countries (18). Building on the primary elements of vulnerability as defined by the Intergovernmental Panel on Climate Change (IPCC) (19) Carley et al. (11) pioneered the application of the vulnerability scoping diagram (VSD) framework from climate change adaptation literature (e.g. (9)) to energy transition analysis. Their study devised a measure for energy transition vulnerability across counties in the United States from the implementation of the renewable portfolio standard. Subsequent studies have expanded this framework to the US fossil fuel communities (20, 21) and European NUTS2 regions (22). While these subnational studies contribute to a more just energy transition within specific nations, a robust, systematic, and transparent method to measure and compare energy transition vulnerability across countries is urgently needed but notably absent.

**Fig. 1.**
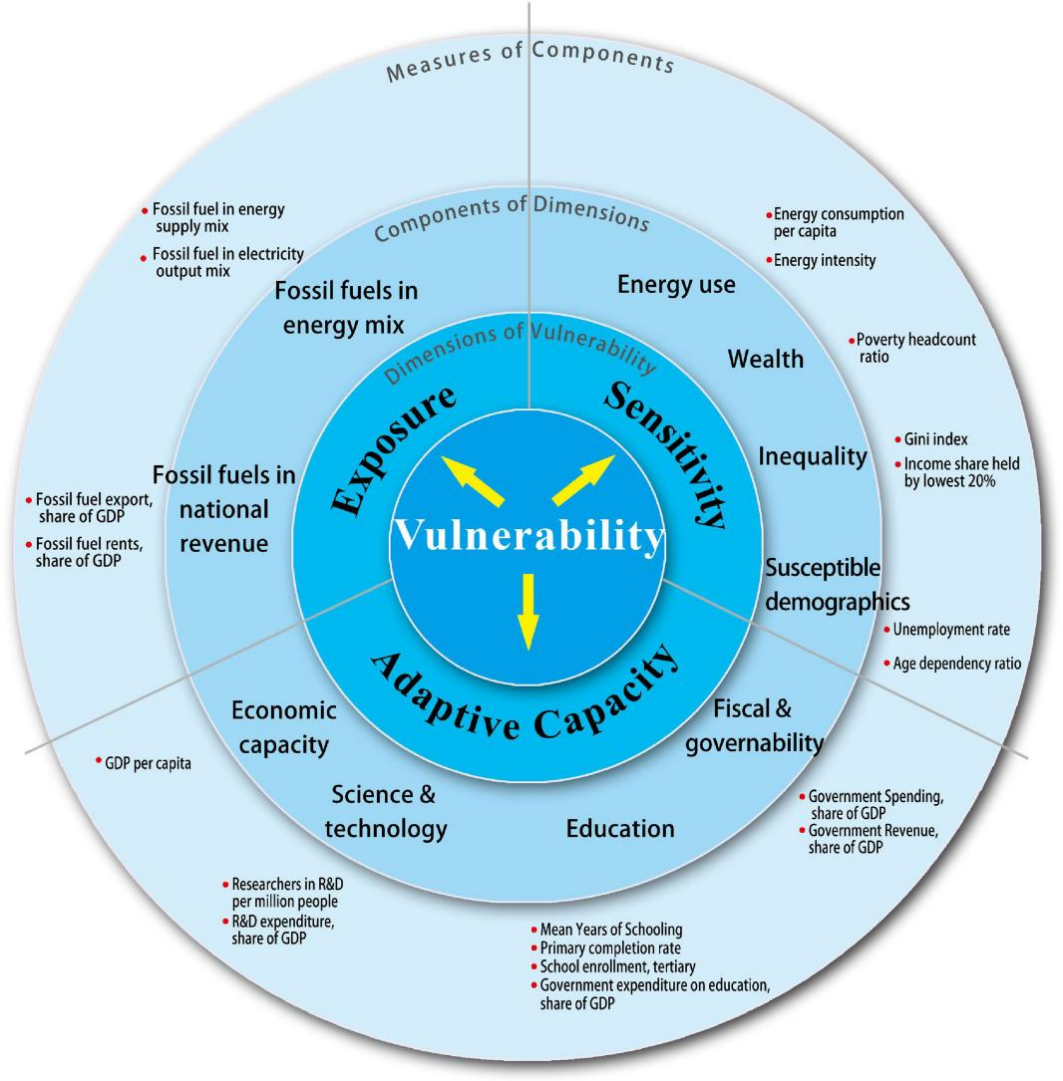
Country-level vulnerability scoping diagram for energy transition: A focus on the events of transition away from fossil fuels. Building on the framework introduced by Carley et al. (11) and the original IPCC definition (19), our study adapts the vulnerability scoping diagram (VSD) method to assess the country-level energy transition vulnerability. Specifically, we define the vulnerability of energy transition as a composite function involving the magnitude of changes required in both the energy and economic systems for the decarbonization transition (exposure), a country's susceptibility to the impacts of these changes (sensitivity), and the capacity of a country to attenuate, cope with, or mitigate adverse effects (adaptive capacity). For each dimension, related concepts (components of dimensions) and measures of components can be identified. Notably, in this study, we examine the vulnerability to events of transition away from fossil fuels—a focal point highlighted in COP28 (2), and define the share of fossil fuels in energy mix and national revenues as key exposure indicators for each country in our VSD conceptual framework. The detailed design of our conceptual framework, including the selection of indicators across all three dimensions, is elaborated in the “Methods” section.

The present study responds to this research need by investigating countries’ past and present status of energy transition vulnerability from the *exposure*, *sensitivity*, and *adaptive capacity* dimensions. In particular, we provide assessments of energy transition vulnerability by quantifying the *energy transition vulnerability index* (ETVI) scores for 135 countries from 2010 to 2020. Countries in this study cover more than 98% of the global Gross Domestic Product (GDP), 92% of the world's population, 93% of the world's energy consumption, and 98% of the world's CO_2_ emissions in 2019 (according to International Energy Agency [IEA] and World Bank data). Figure 1 provides our rendering of the country-level energy transition VSD framework. Our study extends the VSD analysis in Carley et al. (11) to the global scale and adds a dynamic temporal perspective into the examination. The three dimensions of vulnerability align with the original IPCC definition of vulnerability (19). However, the scope of components and corresponding indicators, have been adjusted to accommodate our national-level data and questions of interest. The rationale for indicator selection and the supporting reference for each element in our VSD framework are discussed and presented in “Methods” section.

Like other studies on vulnerability to the energy transition (11, 20–22), we focus on the challenges of the energy transition without denying its opportunities and benefits. In particular, we examine the vulnerability to events of transition away from fossil fuels—a focal point highlighted in COP28 and define the share of fossil fuels in energy mix and national revenues as key exposure indicators for each country in our VSD conceptual framework. The transition away from fossil fuels is pivotal for achieving net-zero emissions but could introduce substantial vulnerabilities due to its extensive economic, social, and infrastructural impacts. This is particularly pronounced in economies heavily dependent on fossil fuels, where the shift impacts energy supply, national revenue, and employment sectors, necessitating substantial adjustments in energy infrastructure and workforce dynamics. A vulnerability perspective is important because early detection of vulnerability can ensure adequate time and preparation for just transition programmes (3, 23). This awareness of challenges and risks enables policymakers to develop targeted strategies and allocate resources effectively to mitigate socioeconomic disparities and manage potential disruptions. By doing so, the transition can be inclusive and maintain broad social and political support, which is essential for achieving a just, orderly, and equitable transition, as emphasized by COP28 (2).

Based on the proposed energy transition vulnerability measurements, this study establishes baselines and offers quantitative insights into the spatiotemporal patterns and trends in energy transition vulnerability. We analyze the evolution of ETVI scores for each country, focusing on the regional heterogeneity in energy transition vulnerabilities between Global North and Global South countries. A specific emphasis is placed on investigating the impact of the COVID-19 pandemic on energy transition vulnerability. Furthermore, this study provides two important applications of proposed energy transition vulnerability measurements, aligning them with the major global sustainable development agenda. Firstly, this study examines the difference in seven climate party groups defined by the United Nations Framework Convention on Climate Change (UNFCCC) in global climate negotiations. Secondly, this study explores the possible synergies between the energy transition resilience and countries’ progress toward the 2030 United Nations Sustainable Development Goals (SDGs). Understanding the interplay between energy transition, climate change negotiations, and SDGs—three global major policy agenda—is pivotal for shaping effective and integrated global policies (24). In turn, this helps in the formulation of strategies that simultaneously address sustainability, equitable climate mitigation, and the achievement of SDGs, ensuring a holistic approach to global challenges. In the climate party group’s analysis, this study connects the status quo of energy transition vulnerability with CO_2_ emission accounts for each country and identifies four types of energy transition status: *Stressful*, *Leapfrog*, *Potential Challenges*, and *Less Painful*. The results help to identify countries that require special assistance from the global community to ensure an inclusive and just energy transition and should assist in the global decarbonization pathways design. In the energy transition resilience-SDGs nexus analysis, the results provide insights into the interventions required to achieve synergies (25–27) and what could be delivered in terms of transition vulnerability if relevant SDGs were to be achieved.

Our study has three key contributions. Firstly, our method and ETVI scores contribute to the global efforts in evaluating countries’ energy transition (28, 29). If appropriately applied and updated, our constructed energy transition vulnerability measures have the potential to guide a just energy transition, supporting nations in achieving their net-zero commitments by 2050. Secondly, the analysis of energy transition vulnerability across countries, including the identification of four types of transition status, serves as a valuable tool for national decision-makers. It helps them pinpoint weaknesses and barriers in their energy transition efforts, while providing international organizations with the means to monitor vulnerabilities and offer support for a more rapid and equitable energy transition. The understanding of the energy transition vulnerability of climate negotiation groups (30) further enriches our understanding of the possible motivations behind these groups, whose actions significantly influence the outcomes of global climate negotiations. Thirdly, the broad impacts of the energy transition entangle the Paris Agreement climate targets (31) with the SDGs (32). Our analysis of the links between energy transition vulnerability and broad measures of social and economic development, such as the delivery on SDG targets, highlights the important synergies between the energy transition and sustainable development.

## Results

The ETVI scores provide an assessment of a country's overall magnitude of energy transition vulnerability, compared with the best global and all-time possible outcome, by summarizing the country's performance from the dimensions of exposure, sensitivity, and adaptive capacity (more details on constructing the ETVI scores, see “Methods” section). The index score signifies a country's position between the least vulnerable (0) and the most vulnerable (100) for adverse impacts of energy transition. That is, a *lower* score means a nation is less vulnerable, and a *higher* score means a nation is more vulnerable. For example, Iceland's overall index score in 2019, 10.73, suggests it is, on average, around 11% points away from the globally best possible outcome in relation to energy transition vulnerability.

**Fig. 2.**
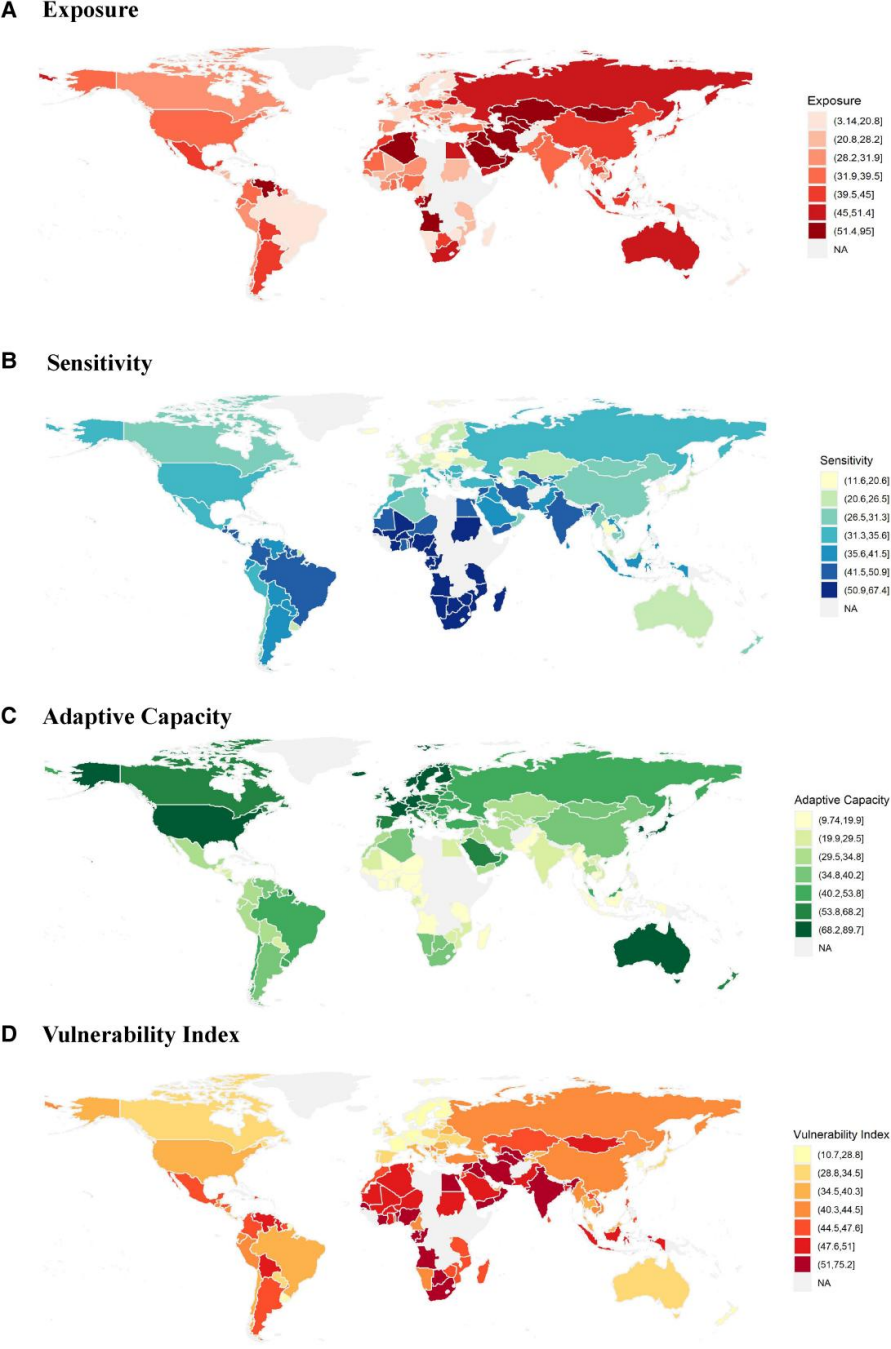
Energy transition vulnerability index (ETVI) scores in 2019. Maps (A–C) show exposure (A), sensitivity (B), and adaptive capacity (C) to energy transitions, using a quantile classification scheme so that each category has an equal number of observations. Map D shows vulnerability scores. A lower vulnerability score means the nation is less vulnerable to the adverse impacts of energy transition. For more details on generating the index, see “Methods” section.

We calculated energy transition vulnerability measures for 135 countries from 2010 to 2020 and reported the 2019 ETVI score (Fig. 2, Table S2) as the baseline status of energy transition vulnerability for each country. By adopting 2019 as the baseline, we avoided both the shocks of COVID-19 and missing data in 2020 for some countries. The impacts of COVID-19 are, nevertheless, discussed in our analyses of time trends of the energy transition vulnerability.

### Energy transition vulnerability across countries

The roster of the least vulnerable countries prominently features economically developed nations (Fig. 2d, Table S2). Among the top 20 countries, all except Uruguay are the Organisation for Economic Co-operation and Development (OECD) member countries. Leading the 2019 vulnerability index scores are three Nordic countries: Iceland, Sweden, and Denmark. The Nordic countries stand out not only for their advanced economic development levels but also for their reduced dependence on fossil energy. Notably, Iceland relies on geothermal energy for 65% of its total primary energy supply, complemented by an additional 20% from hydropower (33). This diversified energy mix positions Iceland as less susceptible to the challenges associated with transitioning away from fossil fuels, a requirement of the global low-carbon energy transition. We emphasize, however, that even economically developed OECD countries may encounter substantial challenges in achieving inclusive energy transitions. For example, OECD countries, such as Australia, Japan, and South Korea, are highly exposed to the transition away from fossil fuels due to the substantial proportion of fossil fuels in their energy mix (Fig. 2a). Further, the European energy crisis of 2021–2022 underscores the energy transition vulnerability of high-income and industrialized countries, such as Germany and the United Kingdom. For instance, sanctions on Russian oil and gas, prompted by the Ukraine crisis, led to a forced transition and a subsequent sharp surge in energy prices in 2022. This increase substantially raised the cost of living, as reflected by the consumer price index for households, exposing the underlying energy transition vulnerability in OECD countries.

Low- and lower middle-income countries tend to be more vulnerable to energy transition (Fig. 2d), which remains inherent in the vulnerability facet of the energy transition process. Assuming a similar level of transition exposure, poorer countries tend to have higher sensitivity (Fig. 2b) and frequently lack adequate economic, fiscal, and technological capacity (Fig. 2c) to adapt to key socioeconomic challenges induced by energy transition. The Republic of the Congo, Angola, Iran, Iraq, and Syria, are identified as the most vulnerable countries in achieving the energy transition. These countries are not only highly dependent on fossil fuels and are highly sensitive to energy transition but also have limited economic and technological strength to adapt to these challenges. For example, Iraq has 98.9% of total energy supply (TES) from fossil fuels in 2019 and fossil fuel revenue contributes to 27.2% of its GDP while its GDP per capita is only $4854 (compared to the world average of $10936). As a result, Iraq is expected to experience significant socioeconomic pressures in the national and international decarbonization process. Overall, owing to the socioeconomic development status, the Global North countries are generally less vulnerable than those in the Global South, indicating the North–South division is also persistent in terms of energy transition vulnerability (Fig. 2d).

Energy-exporting countries exhibit varying levels of vulnerability (Fig. 2d, Table S2). Countries in Middle East can be broadly divided into three types. Firstly, countries like the United Arab Emirates, despite having substantial exposure to fossil fuels, have lower transition sensitivity and possess better adaptive capacity, making them less vulnerable to the energy transition. This aligns with the United Arab Emirates’ status as having the most diversified economy within the Gulf Cooperation Council, marked by significant expansions in service sectors such as tourism, banking, and commerce. Secondly, countries like Iran and Iraq, are characterized by extremely high exposure coupled with limited adaptive capacity, face the greatest transition vulnerability globally. Thirdly, countries such as Saudi Arabia and Kuwait outperform Iran and Iraq but still exhibit high vulnerability to the energy transition compared to most nonenergy-exporting nations. These results underscore the crucial point that “not all energy-exporting countries are alike” during the global low-carbon energy transition process.

### The declining trend of energy transition vulnerability was interrupted by COVID-19

**Fig. 3.**
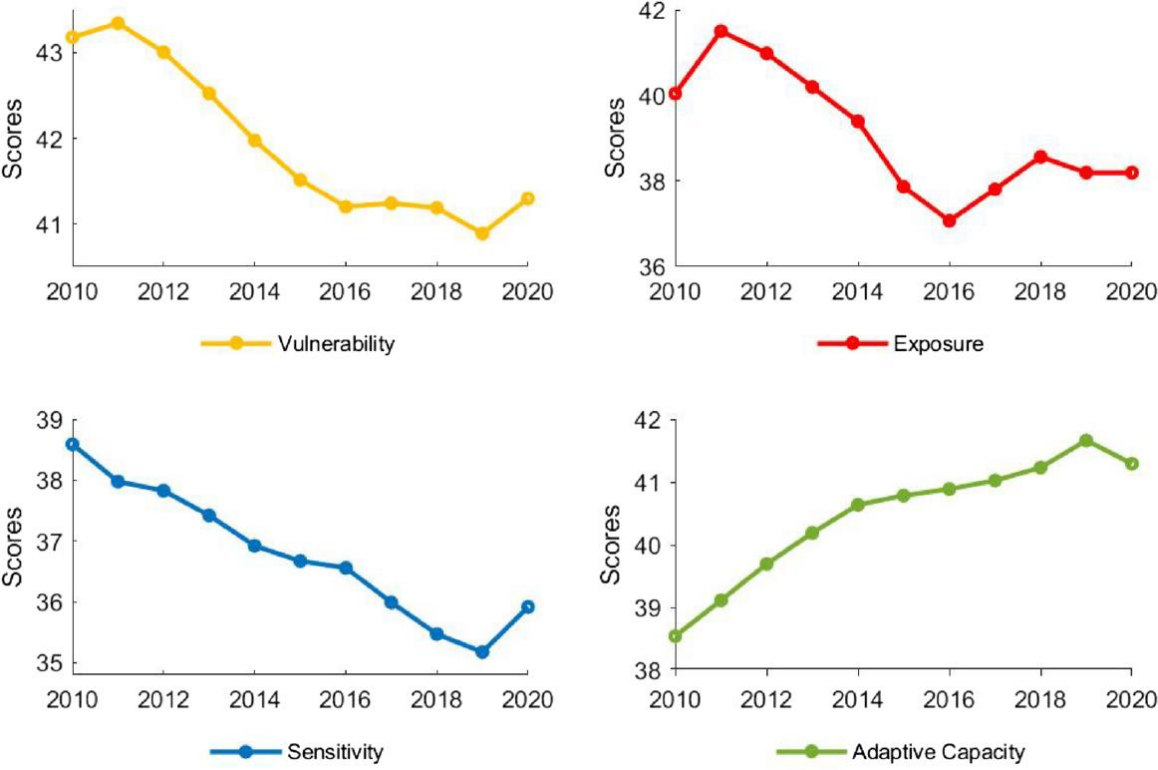
World average ETVI score over time. The world average ETVI score and its three dimensions are measured by equally weighted average across countries. The results are robust according to alternative aggregation methods.

The COVID-19 pandemic interrupted the decade-long trend of declining global average energy transition vulnerability, as depicted in Fig. 3. The reverse trend is largely driven by a surge in sensitivity due mainly to rising poverty and unemployment rates, followed by reduced adaptive capacity (mainly caused by slower GDP growth) and a slightly higher level of the exposure dimension (34). Given the profound impacts of COVID-19 and following the Ukraine crisis, it is not yet possible to definitively determine whether such a trend reversal is temporary. Nevertheless, aggregated exposure scores also show a significant reverse during the 2016–2018 period. This appears to be primarily caused by a surge in global energy prices, which increased fossil energy dependence of energy-exporting countries.

**Fig. 4.**
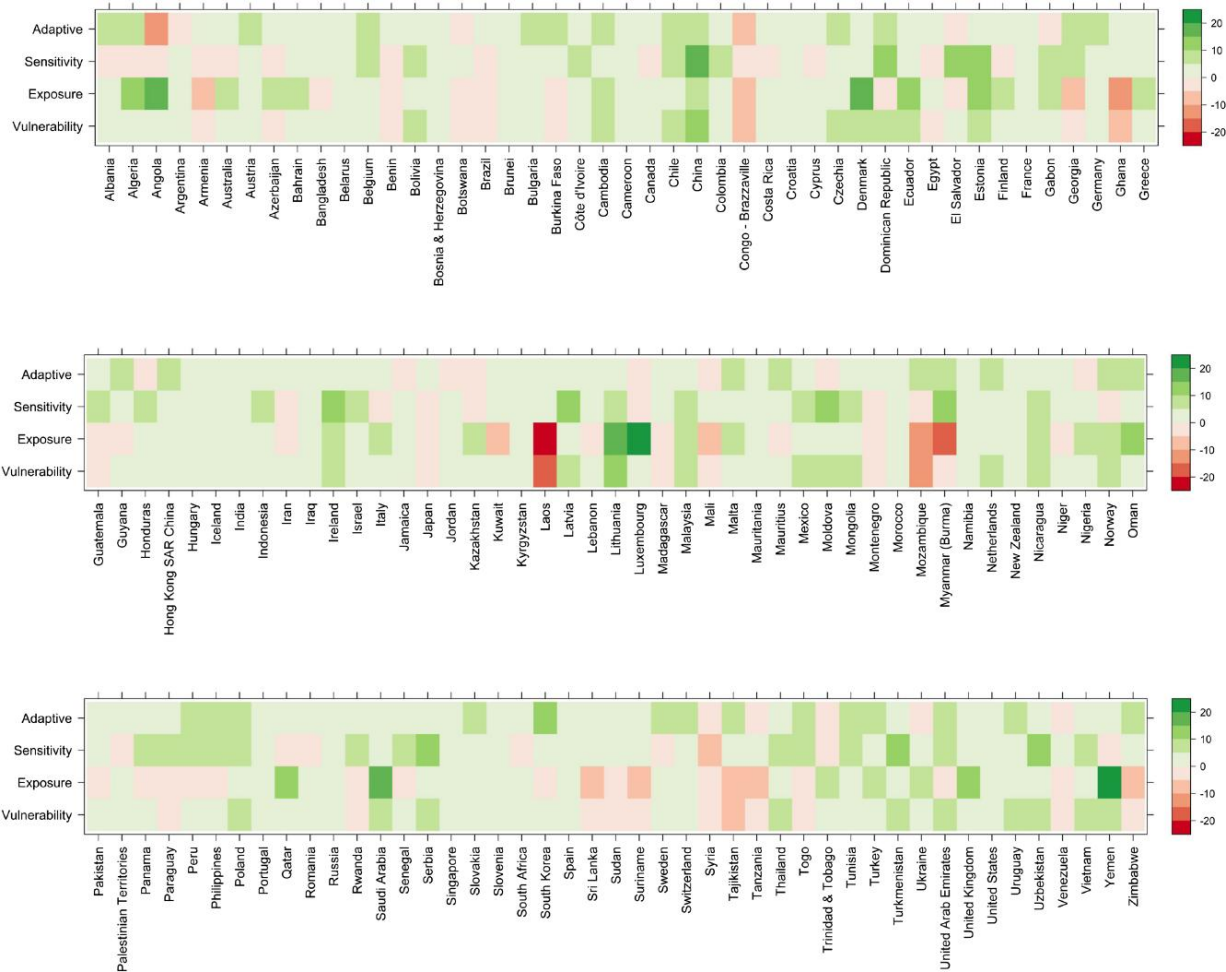
Changes in ETVI scores in the 2010s for each country or region. The color scale shows the changes in ETVI scores and their three dimensions. A positive value indicates an improvement in the score from 2010 to 2019, while a negative value indicates a deterioration in the score over the same period.

In the broader context of decreasing energy transition vulnerability globally, several concerning trends emerge. Notably, our findings reveal that 30 countries, constituting 22% of the assessed nations, witnessed an increase in vulnerability during the study period from 2010 to 2019. Specifically, 43 countries (32%) faced an upturn in exposure to energy transition, another 43 countries (32%) experienced heightened sensitivity, while 16 countries (12%) recorded a decrease in adaptive capacity (Fig. 4).

A desirable convergence in energy transition vulnerability among countries is not yet observed. Those countries with low initial ETVI score in 2010 (indicating low vulnerability) have, as a group, consistently reduced their vulnerability, while those with an inferior vulnerability index score remained in a worse situation (Fig. S2). Lithuania, Estonia, and China recorded the fastest reduction in energy transition vulnerability due to improvements in all three of the vulnerability dimensions. By contrast, Lao PDR, Mozambique, and Ghana had high initial energy transition vulnerability, experienced the worst deterioration of energy transition vulnerability, primarily due to the increased fossil fuel dependence coupling with their economic developments (Fig. 4). This divergence underscores the urgent need for collaborative global efforts to support countries with high energy transition vulnerability, ensuring a more equitable and unified progress toward sustainable energy futures.

### Climate party groups and global decarbonization pathways

Energy transition serves as a crucial strategy to mitigate CO_2_ emissions and combat climate change, while climate party groups represent the substantive common interests of diverse entities in climate negotiations (30). A comprehensive understanding of the energy transition vulnerability across these climate negotiation groups enhances our insights into the determinants of global climate negotiation outcomes, contributing to the formulation of inclusive global decarbonization pathways.

In this section, we move beyond examining the acknowledged responsibility-side of CO_2_ emissions and consider the cost-side of distinct energy transition vulnerabilities across the seven major party groups in international climate change negotiations, as defined by the UNFCCC (for more details on climate party groups, refer to Table S3). Notably, the revealed energy transition vulnerability in this study is likely to be very different from the vulnerability to climate change, which has been widely investigated in the literature (8, 9). Monitoring the vulnerability of climate party groups in the energy transition, together with their CO_2_ emissions, is a critical step that can significantly inform and enhance global climate strategies. This monitoring helps identify countries or country groups that could: (i) enhance their decarbonization commitments at a relatively low socioeconomic cost; (ii) encounter greater challenges in their energy transition; and (iii) require special assistance from the global community to ensure an inclusive and just energy transition.

**Fig. 5.**
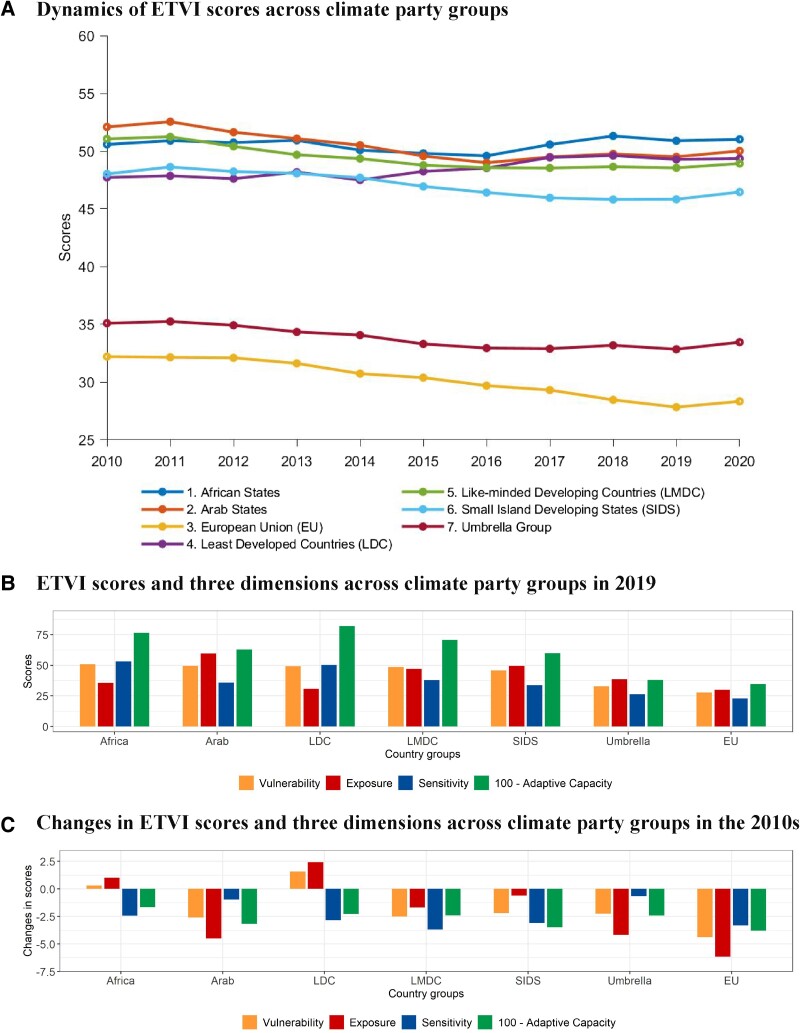
ETVI scores across climate party groups. A) Average ETVI score at climate party group level from 2010 to 2020. B) Average ETVI score and its components for each climate party group in 2019. C) Change in ETVI score and its components for each climate party group between 2010 and 2019.

The ETVI scores exhibit significant heterogeneity across climate party groups (Fig. 5). The European Union (EU) and Umbrella countries, the two most economically developed climate party groups, outperform other groups (Fig. 5a and b), with the EU vulnerability showing the most substantial decline in the 2010s (Fig. 5c). Among the remaining five groups, the Small Island Developing States group (SIDS) consistently maintains the lowest vulnerability scores throughout most of the period. Despite facing relatively high exposure due to their greater reliance on fossil fuels in the energy mix, their vulnerability index score remains much lower than the other four groups, attributed to their comparatively better status in transition sensitivity and adaptive capacity dimensions (Fig. 5b). The African States group and Least Developed Countries group (LDCs) appear to face significant challenges in energy transitions, as their ETVI scores are high and have deteriorated over the past decade, primarily due to increased exposure to fossil fuel dependence (Fig. 5c). In contrast, despite having the highest exposure, countries in Middle East have notably reduced their reliance on fossil fuels over the decade, resulting in improvements in ETVI scores. The Like-minded Developing Countries (LMDCs) have consistently decreased their vulnerability by making relatively balanced improvements in all three vulnerability dimensions, despite starting with a high initial transition vulnerability status.

**Fig. 6.**
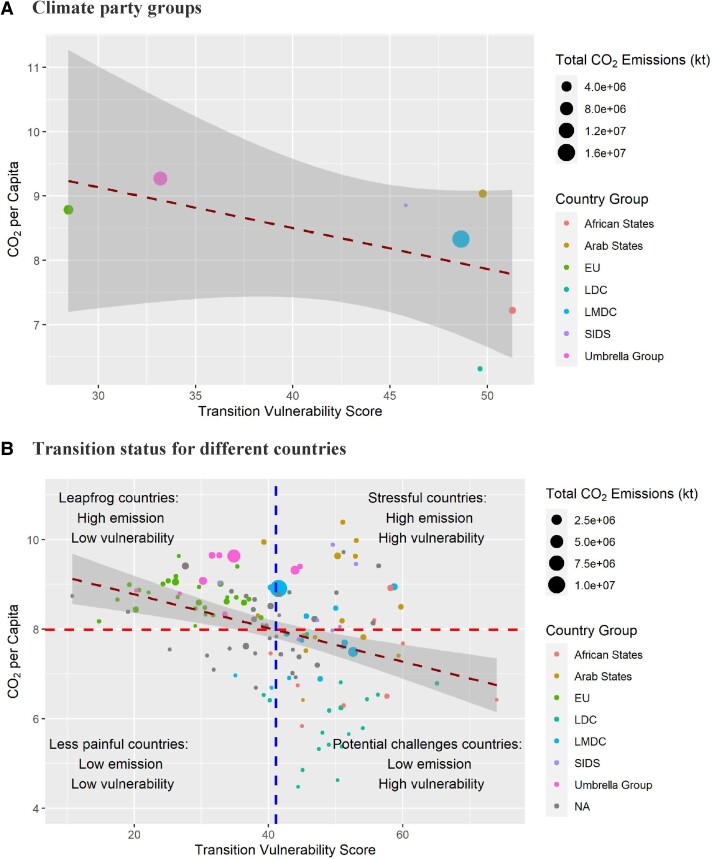
Transition vulnerability and CO_2_ emission in 2019. The relationship between transition vulnerability and CO_2_ emission per capita at A) the climate party group level and B) national level. We report the results in 2019 to reflect the current status of each country. The vertical and horizontal lines are the mean value for *x* and *y* axis variables, respectively. The dark red line stands for the fitted linear curve for the country group and full sample regression with 99% confidence intervals, which shows a negative relationship between energy transition vulnerability and carbon emission. The logarithmic transformation has been applied for CO_2_ emission per capita (kg) data to avoid extreme values.

Figure 6 shows that energy transition vulnerability and CO_2_ emissions per capita have a negative relationship. The results highlight that, besides the responsibility-side of CO_2_ emissions, it is important to account for the cost-side of energy transition vulnerability when designing global decarbonization pathways. Following the practice of the World Economic Forum (WEF) (28), we adapted a bivariate analysis framework and divide all countries into *four* quadrants (transition status) to reflect their relative resilience in energy transition and accounts in global carbon emission patterns: *Stressful*, *Leapfrog*, *Potential challenges*, and *Less painful* (Fig. 6b). The results show that the EU and most Umbrella countries have low vulnerability and high emissions (Quadrant II, Leapfrog countries) and, thus, are more capable of undertaking a faster energy transition in meeting the global decarbonization targets. The majority of the LDC group has high vulnerability scores with relatively low emissions (Quadrant IV, Potential challenges countries). Unfortunately, few countries achieve low vulnerability and low emissions simultaneously (Quadrant III, Less painful countries), which is the most desirable status to meet the dual goals of energy transition and decarbonization. Countries in the LMDC group, in general, have a substantial vulnerability to energy transitions, but their emissions could be either relatively low or high. The Arab States and SIDS group need particular attention as they are mainly located in the least desirable quadrant—a relatively high vulnerability and high emissions (Quadrant I, Stressful countries).

The negative relationship between energy transition vulnerability and CO_2_ emissions per capita is supported by fitting the linear regression model (Fig. 6b), where the slope coefficient is negative and statistically significant (at a significance level of 1%). Our results suggest that the socioeconomic hardships could be mitigated in the global decarbonization process if an appropriate pace is taken according to different transition status across countries (12). For example, in general, the *Leapfrog* countries, such as the United States, Canada, and Australia should be incentivized for faster energy transition as they have the capacity to do so while their CO_2_ emissions is relatively high (on average, 15.3t per capita). However, the *Stressful* countries, such as Saudi Arabia and Iran, the energy transition is urgently needed but should be carefully managed (12). The energy transition should not be too radical for these countries and should be aligned with their socioeconomic developments since they are relatively more vulnerable to the process of energy transitions, and the international assistant are needed in their energy transitions. For the *Potential challenges* countries, their decarbonization policies need not be extremely stiff as they have the lowest CO_2_ emissions per capita but high transition vulnerability. However, since most of *Potential challenges* countries are developing countries, green development path is needed to ensure CO_2_ emissions are not coupled with their socioeconomic developments.

### Synergies between SDGs and the energy transition resilience

The broad impacts of the energy transition entangle the Paris Agreement climate targets (31) with the SDGs (32). While the energy transition plays an essential role in achieving the climate change target of SDG 13, progress toward SDGs may also enhance energy transition resilience and thus show possible synergies. Understanding the intricate relationships between energy transition, climate change goals, and SDGs—the three major global policy agendas—is crucial for shaping effective and integrated global policies.

In this section, we examine these possible synergies and evaluate how actions related to SDGs may impact energy transition vulnerability across countries. That is, we conducted a scenario analysis utilizing five representative indicators from our conceptual framework, which were also directly employed to construct the country-level SDG index in the Sustainable Development Report 2021 (34, 35) (Table 1). Three scenarios were considered: the *global frontier scenario (scenario #1)* assumed all countries’ selected SDG performance are converged to the global top 20% counterparts in the sample; *group frontier scenario*  *(scenario #2)* assumed countries in each climate party group reached their top 20% counterparts within their group; and *group catch-up scenario (scenario #3)* modestly assumed that the countries below the average in their climate party group were able to catch-up with the average of their group. Similar scenarios settings have been recently applied to simulate sustainable developments paths or CO_2_ reduction capacity (e.g. 36–38). The five selected SDGs and their targeted values in each scenario are summarized in Table 1. Note that while our scenario analysis only simulated the partial impacts with five dimensions of selected SDGs, our results nevertheless do illustrate how SDGs and energy transition vulnerability may interact.

**Table 1.** Target values of selected SDGs under scenarios 1, 2, and 3.

Dimension	Target variable	Related SDG	Country group	Scenarios #1 target (World top 20%)	Scenarios #2 target (Top 20% in each group)	Scenarios #3 target (Mean in each group)
Energy use	Energy intensity (toe/1000 USD [2010 PPP])	SDG 7	World	0.08	0.10	0.17
			African	0.08	0.13	0.23
			Arab	0.08	0.12	0.18
			EU	0.08	0.06	0.09
			LDC	0.08	0.12	0.24
			LMDC	0.08	0.12	0.19
			SIDS	0.08	0.07	0.18
			Umbrella	0.08	0.06	0.17
Wealth	Poverty headcount ratio at $3.20/day (%)	SDG 1	World	2.36	8.74	17.60
			African	2.36	18.72	36.86
			Arab	2.36	5.14	9.47
			EU	2.36	2.09	2.54
			LDC	2.36	22.53	51.05
			LMDC	2.36	6.45	12.74
			SIDS	2.36	3.96	7.73
			Umbrella	2.36	2.26	2.79
Inequality	Gini coefficient	SDG 10	World	30.10	33.24	37.60
			African	30.10	38.60	44.81
			Arab	30.10	36.59	39.16
			EU	30.10	26.18	29.74
			LDC	30.10	37.42	41.41
			LMDC	30.10	35.57	39.75
			SIDS	30.10	38.72	42.34
			Umbrella	30.10	26.40	30.91
Science & Technology	Researchers in R&D (per million people)	SDG 9	World	3,862.66	3,114.46	1,952.75
			African	3,862.66	1,638.61	568.52
			Arab	3,862.66	1,791.82	1,050.30
			EU	3,862.66	5,385.03	3,881.23
			LDC	3,862.66	1,722.55	554.92
			LMDC	3,862.66	1,696.72	792.31
			SIDS	3,862.66	2,362.61	1,873.05
			Umbrella	3,862.66	5,530.94	3,882.89
Education	Mean years of schooling (years)	SDG 4	World	12.10	10.19	8.92
			African	12.10	7.68	5.78
			Arab	12.10	9.24	7.53
			EU	12.10	12.69	11.89
			LDC	12.10	6.60	4.14
			LMDC	12.10	9.60	7.67
			SIDS	12.10	10.51	9.45
			Umbrella	12.10	12.90	12.41

Five indicators for related SDGs are suggested by the UN global indicator framework and are used to construct the country-level SDG index in the Sustainable Development Report 2021 (34, 35). We assign each indicator with the same weight when conducting the scenario analysis to avoid the scaling effects. Stated differently, we treat five indicators as equally important, which can also fully represent the corresponding component in our conceptual framework.

**Fig. 7.**
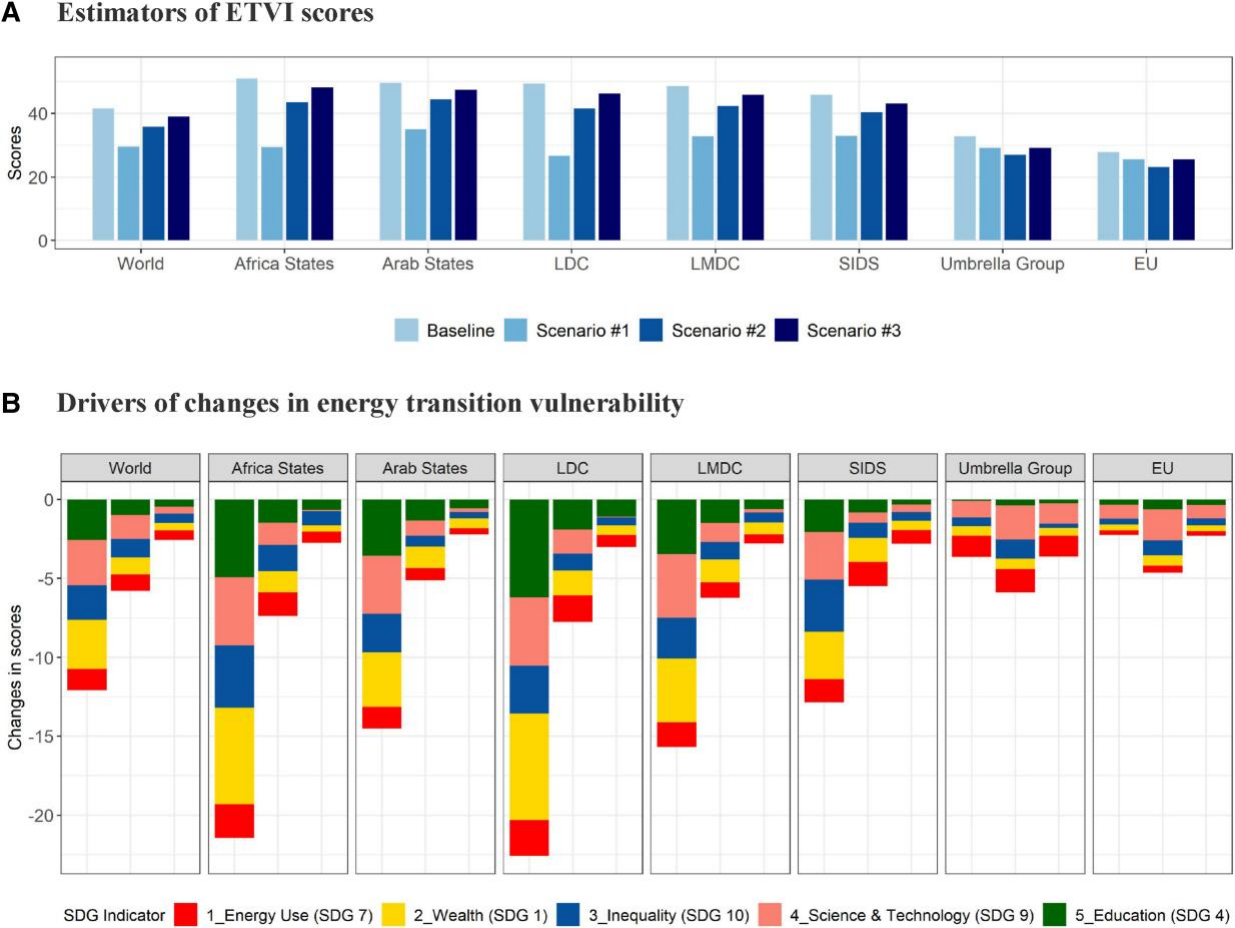
Estimators of ETVI scores in different SDG achievement scenarios. Indicators for related SDGs are suggested by the UN global indicator framework and are used to construct the country-level SDG index in the Sustainable Development Report 2021 (34, 35). We assign each indicator with the same weight when conducting the scenario analysis to avoid the scaling effects. Stated differently, we treat five indicators as equally important, which can also fully represent the corresponding component in our conceptual framework. A) Estimators of ETVI scores. B) Drivers of changes in energy transition vulnerability.

In essence, our findings underscore the synergy between advancing SDGs and a consequential reduction in energy transition vulnerability. The extent of this synergy, however, varies across different scenarios of SDG achievement. Globally, the pursuit of SDGs is projected to lead to a noteworthy reduction in ETVI scores—by 12.3 percentage points (p.p.) and 5.2 p.p. in the global and group frontiers scenarios, respectively (Fig. 7a, World). This improvement significantly surpasses the average reduction in energy transition vulnerability observed during the 2010s, which was 2.2 p.p. Nevertheless, the scenario where lagging countries catch-up to their group average yields a more modest benefit, contributing a 2.5 p.p. reduction. These results highlight the potential for governments to play a more substantial role in achieving SDGs and to reduce their countries’ vulnerability to the energy transition. In the scenario of achieving group frontiers (scenario #2), the least desirable country types characterized by high emissions and high transition vulnerability (*Stressful* countries in Fig. 6) are anticipated to constitute 11% of all countries. This marks a notable reduction from the current level of 19% (Fig. S3). The findings underscore the critical role of comprehensive SDG achievement in mitigating energy transition vulnerabilities globally.

The impact of progress toward SDGs on energy transition vulnerability exhibits heterogeneity among various climate party groups (Fig. 7b). Notably, the attainment of SDGs is projected to have a substantial effect on vulnerability in African countries and LDCs, whereas its impact on the vulnerability of EU and Umbrella countries is comparatively minimal. This discrepancy stems from the high level of socioeconomic development in the EU and Umbrella groups, where further advancements in SDGs exert little direct impact. Furthermore, the drivers of vulnerability reduction vary among climate party groups. For EU group countries, the primary driver of vulnerability reduction is the improvement in Science and Technology Capability (SDG 9). By contrast, for the Umbrella countries, the most significant reduction in transition vulnerability results from advancements in both Science and Technology Capability (SDG 9) and Lower Energy Intensity (SDG 7). Within developing party groups, Poverty Reduction (SDG 1), Reduced Inequality (SDG 10), and Education (SDG 4) emerge as the top three drivers of vulnerability decline. The distinct major drivers identified for different countries provide policymakers with valuable insights into the specific areas they can control to mitigate vulnerability during the energy transition.

## Discussion

To achieve just and inclusive energy transitions, it is imperative to evaluate the vulnerability of countries in this process. However, existing literature and policy frameworks lack comprehensive measures for assessing energy transition vulnerability on a global scale. This research introduces a new framework designed to quantify energy transition vulnerability and conducts a temporal analysis for 135 countries. Our focus is specifically on the events of transition away from fossil fuels, utilizing the percentage of fossil fuels in the energy mix and national revenues as key exposure indicators within our VSD conceptual framework. This emphasis aligns directly with the mandate of COP28 (2), which calls on global efforts to transition away from fossil fuels in energy systems in a just, orderly and equitable manner. The methods employed and the ETVI scores derived from this study can contribute to global initiatives aimed at monitoring energy transitions (18, 29). Furthermore, they can serve as valuable tools to shape policies and collaborative strategies, ultimately mitigating energy transition vulnerability, safeguarding vulnerable nations, and fostering more equitable and effective energy transition policies on both national and international fronts.

Our analyses reveal a noteworthy disparity in energy transition vulnerability between Global North countries (with a more favorable position) and Global South countries (facing a more challenging situation). Additionally, the widening gaps in energy transition vulnerability across countries underscore the necessity for further policy interventions by national governments and potentially the international community. These interventions are essential to safeguard vulnerable countries and populations during their energy transitions. Furthermore, this study provides two important applications of proposed energy transition vulnerability measurements, aligning them with the major global sustainable development agenda. We find significant differences in the dynamics of transition vulnerability across climate negotiation parties defined by the UNFCCC, and identify four energy transition status in achieving the global climate goals. This study also shows the important synergies between the energy transition resilience and 2030 United Nations (UN) SDGs.

The impact of COVID-19 pandemic and Russia–Ukraine war needs close attention due to their significant influence on global energy transitions. While the overall energy transition vulnerability had declined globally over most of the last decade, this study shows there is an interruption in this encouraging trend after 2019 due to the COVID-19 pandemic. Even worse, the COVID-19 pandemic has potentially widened the gaps in the energy transition process among countries (39), with some fossil fuel producers may tend to follow “dirty recovery” paths (40). This situation underscores the need for continuous monitoring and further study of energy transition vulnerability, particularly in the post-pandemic period. The surge in global energy prices due to the Russia–Ukraine war presents a complex scenario where fossil fuel exporting countries face increased exposure, while fossil fuel importing countries might be forced to expedite their energy transition process. Special attention is needed for low-income countries, which are more vulnerable to the surge in energy prices and have limited financial and technological resources to mitigate the negative impacts.

Global decarbonization and energy transition pathways are more likely to succeed if they are inclusive, gradual, and tailored to the specific needs of each nation (12). While energy transition plays an essential role in achieving climate targets, overly ambitious energy transition pathways could backfire, especially for those more vulnerable countries. This is because political support can be lost if adverse effects like energy price hikes or supply disruptions can be linked to, or blamed on, the energy transition (even if incorrectly). For example, while the high carbon emissions per capita suggests that the *Stressful* countries (Fig. 6) might need to set faster transitional pathways, their high vulnerability suggests that their transition steps should be carefully considered. By contrast, the *Leapfrog* countries, with high emissions and low vulnerability, could accelerate their energy transition process due to their high adaptive capacities. For countries with low emissions, i.e. *Potential challenges* countries, the focus should be on sustainable economic growth, while promoting energy efficiency and green technologies to minimize emissions during the development. Indeed, continuous socioeconomic development of developing countries is a key means to achieve just and inclusive energy transitions as a principle of the Paris Agreement (31) and is needed to create a fair and cooperative climate regime (41).

Based on these findings, energy transition policies can be more effectively designed to support a fair and efficient global energy transition, while responding to the diverse challenges and adaptive capacities of different countries. Developing countries, which are often the most vulnerable during the energy transition, have limited national capacity to cope with potential socioeconomic hardships triggered by this process and generally have low present and historical emission liabilities. Collaborative efforts from the international community in financing, technology support, and institutional building are crucial for managing vulnerability during the energy transition in these countries (42). The Loss and Damage Fund (43), initiated in COP28, should broaden its scope to include assistance specifically tailored for vulnerable countries in the context of energy transition. Increased investment (44) and additional initiatives, such as international climate finance, carbon pricing revenue redistribution, and promoting clean energy technology transfer, are conducive to facilitate a just and inclusive low-carbon transition, particularly for these vulnerable countries (45). Moreover, regulation is necessary to ensure that developing countries achieve a sufficient level of consumption for “decent living energy” without overshooting necessary limits (46). A case study shows that decent living standards can be maintained in India, Brazil, and South Africa using approximately 90% less per capita energy than is currently consumed in affluent nations (47). For developed countries, given the high and rising inequality in emission levels across their populations, carbon pricing and revenue redistribution can incentivize progress toward a just energy transition. Recent evidence shows that the poorest 50% of the population in North America emit fewer than 10 tons of carbon dioxide equivalents annually, while the top 10% emit around 69 tons (48). Carbon taxation methods and revenue recycling mechanisms are essential tools for curbing carbon emissions and improving the fairness of climate policy (49). Tax revenue can be used to promote a low-carbon transition or be redistributed to low-income households. A recent investigation focused on carbon taxation levied on luxury goods suggests that the resulting tax revenue could represent 1 to 4% of the total GDP in high-income countries (50). Moreover, it has the potential to contribute to the mitigation of approximately 100 Gt of carbon dioxide equivalents by 2050, which is equivalent to 2.67 times the global total energy-related CO_2_ emissions in 2023 (50, 51).

The results of this study also partly contribute to the debates on “green growth” vs. “degrowth” in sustainable development studies. Although the green growth concept is widely embraced by international institutions like the World Bank, and the OECD, its feasibility are questioned (52). For more than half a century, global increases in wealth, represented by burgeoning consumption have escalated environmental impacts at a pace that far exceeds reductions achieved through technological improvements (53). Without limit of growth or consumption in the future, technological solutions for mitigating environmental impact will face increasing pressure and eventually impossible because they will need to not only reduce impacts but also offset the effects of increasing consumption and affluence (52). This leads to the argument that, for the sake of sustainability, economic growth should be intentionally slowed, halted, or even temporarily reversed (referred to as degrowth), for examples, discussed in references (54–59). Our study contributes to this debate from the perspective of vulnerability in energy transitions, suggesting that economic growth can enhance adaptive capacities, thereby mitigating social vulnerability during these transitions and making decarbonization politically feasible. However, it also underscores that improvements in energy transition vulnerability can be achieved through nongrowth measures. These include strategies grouped under the “Sensitivity” dimension, such as reducing inequality and redistributing resources to disadvantaged groups. It is important to note that while we emphasize the significance of growth in achieving an inclusive and politically feasible decarbonization process, attention must also be directed toward promoting sufficiency-oriented lifestyles and curbing overconsumption, as well as addressing widening inequalities during the pursuit of green growth (57, 60–62).

While our study represents the first attempt to measure the countries’ vulnerability in transition to a low-carbon future, it, of course, has several limitations. Firstly, our study builds a valid, consistent, and transparent index system that captures the key aspects of the energy transition vulnerability across countries, which is comparable to the work of energy transition index of World Economic Forum (18) and energy trilemma index of World Energy Council (29). The proposed index system in this article could be further refined when applied to assess energy transition vulnerability in specific countries, considering their unique features. Secondly, while this study focuses on the events of transition away from fossil fuels, the investigations on other shocks required by global low-carbon transition are of particular interest for future studies. Thirdly, while our study focuses on the challenges posed by the energy transition, we do not intend to disregard the opportunities it presents. Further consideration of what factors may benefit from the energy transition would also be a useful extension of our work.

## Methods

### Data

This study provides a comprehensive global analysis of energy transition vulnerability. We collected the energy data from the World Energy Balance Table of the IEA, socioeconomic and environmental data from the World Development Indicators of the World Bank and the Demographic and Social Statistics of UN Statistics Division. Our data covers 135 economies, which represent more than 98% of the world's GDP, 92% of the world's population, 93% of the world's energy consumption, and 98% of the world's emissions in 2019, according to the World Bank. The summary statistics of indicators that are used to evaluate the ETVI index of 135 economies is shown in Table S1.

### Country-level vulnerability scoping diagram for energy transition: a focus on the events of transition away from fossil fuels

According to the IPCC, vulnerability is defined as “the propensity or predisposition to be adversely affected,” which “encompasses a variety of concepts and elements including sensitivity or susceptibility to harm and lack of capacity to cope and adapt” (6). A recent review shows various frameworks in the literature that qualify or quantify the vulnerability from climate change-related disasters, and these frameworks are diversified across disciplines (11). Based on the IPCC definition, the vulnerability scoping diagram (VSD) unifies the diversified conceptions of vulnerability and defines vulnerability from three dimensions: exposure, sensitivity, and adaptive capacity (9). The VSD has been used primarily in the context of natural hazards, disaster management, and climate change adaptation (63, 64), and later extended to the US energy transition (11).

Our conceptual framework for assessing national energy transition vulnerability (Fig. 1) was developed from a pioneer study of Carley et al. (11). Carley et al. (11) adapted the VSD conceptual framework to a social science setting and investigated energy transition vulnerability of the US counties. This study extends Carley et al.'s (11) analysis to the global scale and adds a temporal dynamic perspective into the examination. Specifically, our focus is on the events of transitioning away from fossil fuels—a focal point highlighted in COP28, defining the share of fossil fuels in the energy mix and national revenues as key exposure indicators for each country in our VSD conceptual framework. The focus on transitioning away from fossil fuels is particularly relevant when considering the vulnerability associated with the energy transition, as it tends to be the aspect that generates the most significant concerns and challenges. While other facets of the energy transition may present opportunities and benefits, such as technological advancements and economic diversification in renewable energy sectors, the transition away from fossil fuels directly challenges established economic and social structures, leading to heightened vulnerability (65, 66).

In summary, our VSD conceptual framework comprises three dimensions—exposure, sensitivity, and adaptive capacity—each containing at least two components, further measured by several indicators. Energy transition vulnerability is defined as a function of the underlying magnitude of changes in the energy system required by the transition away from fossil fuels for each country (exposure); the susceptibility of a country to the impacts of these changes (sensitivity); and the capability of a country to attenuate, cope with, or mitigate the negative effects (adaptive capacity). While drawing upon the groundwork laid by Carley et al. (11), the scope of components, and corresponding indicators, has been adjusted to accommodate our national-level data and questions of interest. We selected indicators that capture the critical factors necessary to describe or proxy the three dimensions of energy transition vulnerability as defined. The inclusion of each indicator was carefully validated based on existing research. Detailed discussions regarding the selection of each indicator are provided below.

Regarding the exposure dimension, we focus on the events of transition away from fossil fuels, which aligns directly with the mandate of COP28 (2). Note that, similar to the rise of energy price caused by renewable portfolio standard policy in Carley et al. (11), a transition away from fossil fuels can be regarded as a common policy event imposed on all the countries to meet the global carbon neutrality. The exposure dimension is evaluated by two primary components related to transition away from fossil fuels: the share of fossil fuels in the energy mix and the contribution of fossil fuels to a country's revenue (67). Countries with a higher share of fossil fuels in their energy and electricity generation mix are considered more exposed and, therefore, more vulnerable, given that they must undergo a substantial shift in their current energy sector and economic structures. Consequently, these countries will experience more direct impacts during the process of transition away from fossil fuels. For instance, Iceland, with only 11.1% of total energy supply from fossil fuels in 2021 (IEA), contrasts starkly with China (87.2%) and the United States (81.7%). It is evident that China and the United States would face more exposure compared to Iceland in the process of transition away from fossil fuels. Similarly, countries that derive a larger share of rents or export revenues from the fossil fuels sector are also considered more exposed. These economies will encounter relatively more socioeconomic challenges, as a higher proportion of revenue will be lost due to the transition away from fossil fuels in the energy transition (68, 69).

Countries are likely to exhibit varying levels of vulnerability even when exposed to similar impacts from the energy transition. This variation arises because the country's vulnerability in energy transition is not solely dependent on exposure; it also considers a country's sensitivity to exposure and its adaptive capacity to mitigate the associated risks. The sensitivity dimension measures a country's susceptibility to the impacts of the energy transition and economic structural changes, and it is expected to be positively correlated with the energy transition vulnerability. The sensitivity dimension is assessed through critical components related to the energy transition, drawing from existing literature and policy discussions. Specifically, for a given a level of exposure, countries with higher energy consumption (64, 70), increased poverty ratios (71–74), greater inequality (75, 76), and a higher proportion of susceptible demographics (73, 77–79) are considered more sensitive, and consequently, more vulnerable to the energy transition. Our broad measurements of the sensitivity dimension align with counterparts in Carley et al. (11) and are consistent with commonly used indicators for measuring social vulnerability, as seen in Cutter and Finch (80) and Flanagan et al. (81).

The adaptive capacity dimension assesses a country's ability to diminish, cope with, or alleviate negative impacts, showing a negative correlation with country's energy transition vulnerability. Evaluation of the adaptive capacity dimension involves critical components related to the energy transition, drawing from existing literature and policy discussions. Specifically, a country with lower economic development levels (3, 11, 82, 83), reduced scientific and technological capabilities (84–86), lower educational attainment (69, 87–89), and fewer fiscal and financial resources (90–92) will encounter more challenges in attenuating, coping with, or mitigating negative impacts compared to countries with higher levels of these factors when facing similar exposure levels.

Our broader definition of energy transition vulnerability could be an advantage over the framework in Carley et al. (11) for the US counties due to the rich country-level and temporal data provided by reputable international organizations such as the World Bank and IEA. Notably, as in Carley et al. (11), our primary focus is on evaluating the challenges posed by the energy transition, with no intention to diminish the numerous significant benefits and development opportunities it brings about. Exploring factors poised to benefit from the energy transition could serve as a valuable extension of our work.

### Quantitative method: a composite index analysis

To operationalize the VSD with data and extract an ETVI score for a specific country, we adopted the geometric mean of three normalized and arithmetic averaged dimensional indices of exposure, sensitivity, and adaptive capacity. In particular, we applied the following equation:


$$V=\sqrt[{3}]{\left(\frac{1}{I}\sum_{i=1}^{I}E_{i}\right)*\left(\frac{1}{J}\sum_{\;j=1}^{J}S_{j}\right)*\left(100-\frac{1}{K}\sum_{k=1}^{K}A_{k}\right)},\;$$

Where *V* is the vulnerability score, *E* is an assessment of exposure with *i* components associated with energy transition, *S* is an assessment of the sensitivity with *j* components, and *A* is an assessment of adaptive capacity with *k* components.

The geometric mean method has been utilized by international organizations to generate the cross-national comparable index, e.g. Human Development Index and SDG index published by the UN. Following the way of the UN in constructing the SDG index, within dimensions, we arithmetically average across each component of exposure, as well as the sources of sensitivity and adaptive capacity. Within each component, each measure is equally weighted since there is no a priori reason to give one measure greater weight than another (11, 34). The standard min–max method has been adopted to normalize the original data, with top and bottom 2.5th-percentile performer as upper and lower bounds for the baseline results (Table S1). The 2.5th-percentile has been used by previous studies (e.g. (34, 93)) to minimize the potential effects of skewed data distributions on the standardized values during normalization. The normalized scores can be used to evaluate relative performance over time and space toward achieving energy transition resilience. For example, if for a particular ETVI indicator a country lagged behind all the other countries in both 2010 and 2019, but improved over time, its score for that ETVI indicator in 2019 would be greater than its score in 2010, but in both years, its score would be lower than that of the other countries. We also experimented with the alternative upper and lower bounds, such as the 1st and 99th percentile, 5th and 95th percentile, which showed the robust results (Fig. S1).

In our study, beyond the standard method to conduct composite index analysis, the geometric mean of three dimensions also has a clear economic interpretation. More specifically, *S* and *A* are multiplied by *E*, because the set of sensitivity and adaptive capacity measures, respectively, are specific to an exposure. Stated differently, the sensitivity and adaptive capacity only matters if a nation is exposed to a certain type of changes required by the global low-carbon energy transition, for example the transition away from fossil fuels considered in this study. Meanwhile, the same level of exposure will have different negative impacts among countries with different levels of sensitivity. The product of exposure and sensitivity measures the magnitude of direct vulnerability given the energy transition. This direct impact could be mitigated or discounted by the adaptive capacity of each country, which yields an overall assessment of the energy transition vulnerability highlighted in this study.

### Sensitivity analysis

The robustness of the ETVI scores were analyzed by taking uncertainty factors into consideration and conducting a sensitivity analysis. Different scenarios were tested to identify the composite index's level of sensitivity to the change in parameters—different upper and lower bounds, weighting schemes, aggregation methods, and a successive exclusion of indicators. The resulting variation of countries’ scores and rankings is depicted in Fig. S1. Overall, our results appear robust to these alternative ways to construct the index. (For more details, see Supplementary Note S3.)

## Supplementary Material

pgae427_Supplementary_Data

## Data Availability

The energy data were retrieved from the International Energy Agency (IEA)'s World Energy Balance Table (https://www.iea.org/reports/world-energy-balances-overview). The socioeconomic and environmental data were retrieved from the World Bank's World Development Indicators (https://databank.worldbank.org/source/world-development-indicators) and Demographic and Social Statistics of UN Statistics Division (https://unstats.un.org/unsd/demographic–social). The climate party group information was accessed from the UNFCCC (https://unfccc.int/process-and-meetings/parties-non-party-stakeholders/parties/party-groupings). All study data, including the generated ETVI scores, are available in the public repository (https://github.com/shentroy/Energy-Transition-Vulnerability-Index-ETVI-scores).

## Preprints

This manuscript was posted on a preprint at: https://doi.org/10.21203/rs.3.rs-1440213/v2.
