# Supplementary material for: Quantifying energy transition vulnerability helps more just and inclusive decarbonization
Source: PNAS Nexus. 2024 Oct 22;3(10):pgae427. doi: 10.1093/pnasnexus/pgae427 (PMC11495377; doi:10.1093/pnasnexus/pgae427)
Supplement: pgae427_Supplementary_Data [file pgae427_supplementary_data.pdf]

Supplementary information

Supplementary note 1: Data

Table S1. Descriptive statistics

Dimensions	Components	Measurements	Mean	Std	Upper bound	Lower bound
**Exposure**	Fossil fuels in energy mix	Fossil fuel in energy supply mix (%)	66.95	27.28	99.99	0.00
		Fossil fuel in electricity output mix (%)	60.33	33.32	100.00	0.03
	Fossil fuels in national revenue	Fossil fuel export, share of GDP (%)	6.81	13.05	49.46	0.00
		Fossil fuel rents, share of GDP (%)	5.46	11.34	42.08	0.00
**Sensitivity**	Energy dependence	Energy consumption (toe per capita)	0.33	0.25	0.94	0.07
		Energy intensity (toe per thousand 2015 USD)	0.21	0.17	0.65	0.05
	Wealth	Poverty rate ($3.20 a day, % of population)	22.47	27.47	85.51	0.01
	Inequality	Gini index	38.52	8.16	55.80	24.80
		Bottom 20% income share (%)	6.64	1.80	9.80	2.90
	Susceptible demographics	Unemployment rate (%)	7.67	5.51	22.08	0.75
		Age dependency ratio (%)	58.70	17.50	98.22	32.32
**Adaptive capacity**	Economic capability	GDP per capita (PPP, constant 2017 international $)	21305.02	20703.14	71496.60	1400.80
	Science & technology	R&D expenditure, share of GDP (%)	0.80	0.85	3.25	0.04
		Researchers in R&D (per million people)	1471.30	1727.78	6375.55	17.08
	Education	Mean Years of Schooling (years)	8.46	3.04	12.90	2.20
		Education expenditure, share of GDP (%)	4.35	1.50	7.59	1.78
		Primary completion rate (% of relevant age group)	89.38	16.56	109.14	44.69
		School enrollment rate, tertiary (% gross)	39.05	28.08	94.78	2.70
	Fiscal & governability	Government spending, share of GDP (%)	15.63	4.92	25.74	6.27
		Government revenue, share of GDP (%)	25.66	9.91	44.79	9.76

Notes: Data are nation-level data from 2010 to 2020, covering 135 economies. We collect the energy data from the World Energy Balance Table of the International Energy Agency (IEA), and socio-economic and environmental data from the World Development Indicators of the World Bank Demographic and Social Statistics of United Nations Statistics Division, which are reputed global data providers. These economies cover more than 97.81% of the global GDP, 92.16% (7.08 billion) of the world's population, 92.96% of world's energy consumption and 97.97% of world's emissions in 2019, according to our data sources. For further discussions and supporting literature on the index framework, see **Methods**.

Supplementary note 2: ETVI score of 135 countries or regions in 2019

Table S2. ETVI score of 135 countries or regions in 2019

Rank	Code	Vulnerability	Exposure	Sensitivity	Adaptive capacity	Total CO_2 (10^3 t)	CO_2 per capita (t)
1	Iceland	10.73	3.14	20.40	80.68	2200	6.24
2	Sweden	14.35	8.50	26.41	86.84	36000	3.54
3	Denmark	16.00	20.72	19.22	89.70	33380	5.76
4	Finland	18.39	16.33	24.32	84.33	44360	8.04
5	Norway	18.92	31.85	19.08	88.86	37350	7.03
6	Switzerland	18.98	12.48	19.58	72.02	37480	4.40
7	France	20.22	14.60	22.66	75.02	309960	4.62
8	Austria	20.75	22.97	23.11	83.18	63180	7.15
9	Slovenia	21.73	25.26	13.45	69.79	14050	6.77
10	Belgium	23.26	30.31	19.28	78.45	93470	8.18
11	Netherlands	24.43	46.52	15.98	80.40	151170	8.77
12	Uruguay	25.55	10.93	26.44	42.29	6520	1.89
13	Germany	25.55	31.65	21.69	75.71	709540	8.56
14	Czechia	25.72	31.92	15.13	64.78	102480	9.64
15	Luxembourg	25.84	24.06	24.26	70.45	9320	15.33
16	Slovakia	26.01	22.18	17.73	55.23	33000	6.06
17	South Korea	26.52	40.02	17.95	74.04	630870	12.22
18	New Zealand	27.01	19.90	29.60	66.54	32210	6.57
19	Ireland	28.73	37.12	19.82	67.77	37110	7.62
20	Hungary	28.74	27.50	20.58	58.03	46390	4.75
21	Malta	28.92	49.33	11.56	57.59	1550	3.20
22	United Kingdom	29.08	31.54	24.63	68.33	358800	5.40

23	Portugal	29.83	30.84	21.17	59.33	49780	4.84
24	Costa Rica	29.91	12.58	35.82	40.63	8260	1.65
25	Japan	30.12	40.37	22.24	69.57	1106150	8.74
26	Lithuania	30.62	21.81	30.28	56.53	11590	4.14
27	Canada	31.23	28.95	29.62	64.47	574400	15.50
28	Croatia	31.72	27.13	24.71	52.42	16580	4.06
29	Paraguay	31.87	10.55	39.15	21.59	8420	1.21
30	Poland	32.00	43.67	16.87	55.50	312740	8.24
31	Singapore	32.26	46.99	23.79	69.98	47360	8.40
32	Australia	32.26	47.67	22.72	68.99	386620	15.48
33	Cyprus	32.36	47.38	16.40	56.41	7230	6.08
34	Ukraine	32.49	28.05	22.37	45.35	185370	4.15
35	Spain	33.24	29.01	29.69	57.35	258340	5.52
36	Estonia	33.33	40.05	25.07	63.11	16000	12.10
37	Latvia	33.47	27.60	30.39	55.31	7630	3.96
38	Albania	33.55	15.77	34.43	30.42	5560	1.94
39	Israel	34.43	48.85	26.56	68.54	61970	6.98
40	United States	34.52	37.26	35.59	68.99	4981300	15.24
41	Greece	34.67	36.58	27.96	59.26	65290	6.08
42	Italy	35.97	35.05	32.96	59.73	324850	5.38
43	Belarus	36.11	50.89	17.43	46.93	59310	6.25
44	Bulgaria	36.19	29.34	31.49	48.71	41130	5.85
45	El Salvador	36.29	21.27	29.79	24.60	6810	1.06
46	Brazil	36.38	18.81	45.67	43.95	427710	2.04
47	Kyrgyzstan	36.40	20.07	37.59	36.10	11000	1.74
48	Thailand	36.55	41.35	17.51	32.58	257860	3.71

49	Hong Kong SAR China	37.65	47.91	27.29	59.18	42550	5.80
50	Chile	37.82	31.63	28.62	40.25	86620	4.62
51	Romania	38.09	28.61	33.02	41.49	74880	3.85
52	Lebanon	38.19	47.84	17.33	32.81	27710	4.04
53	Tajikistan	38.48	19.40	41.46	29.19	7330	0.81
54	Mauritius	38.79	41.09	21.85	34.98	4130	3.26
55	Moldova	38.86	43.75	21.34	37.13	8590	3.17
56	United Arab Emirates	38.96	83.02	14.61	51.25	200300	20.80
57	Azerbaijan	39.73	74.07	13.41	36.87	32020	3.22
58	Malaysia	39.76	51.00	20.79	40.71	239620	7.60
59	Serbia	40.43	40.13	31.62	47.91	45540	6.52
60	Turkey	40.53	35.17	35.26	46.32	412970	5.02
61	Namibia	40.58	17.14	65.26	40.24	4250	1.74
62	China	40.77	39.70	27.74	38.44	10313460	7.41
63	Cambodia	40.77	25.09	31.07	13.04	11160	0.69
64	Myanmar (Burma)	40.85	30.11	28.07	19.36	32520	0.61
65	Nicaragua	40.93	21.24	42.26	23.60	5210	0.81
66	Peru	41.17	29.72	34.63	32.21	54280	1.70
67	Montenegro	41.84	28.93	42.22	40.02	2520	4.05
68	Bosnia & Herzegovina	41.93	37.02	32.76	39.21	22540	6.78
69	Georgia	42.01	25.35	45.15	35.24	9460	2.54
70	Armenia	42.43	28.71	39.21	32.14	5550	1.88
71	Russia	43.16	50.34	34.43	53.60	1607550	11.13
72	Vietnam	43.53	39.67	27.41	24.13	257860	2.70
73	Cameroon	43.83	18.84	55.38	19.29	8620	0.34
74	Rwanda	44.06	21.49	51.00	21.95	1080	0.09

75	Panama	44.11	32.68	37.02	29.06	10140	2.43
76	Guatemala	44.20	20.33	49.91	14.91	18210	1.11
77	Ecuador	44.50	34.18	37.64	31.49	39530	2.31
78	Sri Lanka	44.66	31.04	35.71	19.64	21630	1.00
79	Dominican Republic	44.89	44.83	29.81	32.30	25120	2.36
80	Tunisia	44.97	47.50	33.03	42.03	29980	2.59
81	Madagascar	44.97	17.52	58.62	11.45	3370	0.13
82	Palestinian Territories	45.06	36.57	41.58	39.85	2960	0.61
83	Morocco	45.60	42.41	33.78	33.82	66680	1.85
84	Laos	45.61	30.95	39.00	21.37	18790	2.66
85	Argentina	45.68	40.37	39.39	40.06	177410	3.99
86	Kazakhstan	45.84	63.14	22.17	31.21	220450	12.06
87	Colombia	45.96	32.19	44.73	32.58	79490	1.60
88	Zimbabwe	45.98	20.41	66.59	28.48	12270	0.85
89	Honduras	46.13	24.87	50.53	21.89	9770	1.02
90	Togo	46.16	20.63	59.97	20.50	2260	0.29
91	Jordan	46.66	44.91	31.33	27.80	24700	2.48
92	Mexico	46.68	43.50	33.58	30.36	472140	3.74
93	Philippines	47.10	36.52	37.34	23.38	142240	1.33
94	Tanzania	47.14	20.85	56.68	11.34	11580	0.21
95	Suriname	47.29	38.78	43.62	37.49	2080	3.61
96	Mozambique	47.41	21.67	66.35	25.87	6640	0.23
97	Pakistan	47.85	33.18	40.25	17.94	208370	0.98
98	Mali	48.18	23.55	54.81	13.33	5620	0.29
99	Mongolia	48.75	59.11	31.23	37.23	21320	6.73
100	Bolivia	48.87	44.98	37.13	30.12	22710	2.00

101	Sudan	49.11	24.88	52.73	9.74	20200	0.48
102	Bahrain	49.69	59.87	35.80	42.74	30750	19.59
103	Jamaica	49.76	48.87	36.85	31.60	8510	2.90
104	Brunei	49.79	84.76	31.75	54.14	7140	16.64
105	Saudi Arabia	49.81	73.63	38.45	56.36	514600	15.27
106	Venezuela	49.82	52.94	37.44	37.60	138160	4.78
107	Qatar	49.93	82.20	32.07	52.79	90170	32.42
108	Niger	50.27	30.84	50.44	18.34	2290	0.10
109	Burkina Faso	50.62	29.68	51.91	15.81	4270	0.22
110	Algeria	50.64	66.40	30.49	35.85	151670	3.59
111	Bangladesh	50.65	45.00	33.55	13.90	82760	0.51
112	Mauritania	50.70	38.21	44.00	22.49	4000	0.91
113	Indonesia	50.92	42.15	38.78	19.21	583110	2.18
114	Ghana	50.96	34.48	46.44	17.33	16110	0.54
115	Guyana	50.97	47.63	43.69	36.35	2440	3.13
116	Cote d’Ivoire	51.05	28.23	55.68	15.38	10250	0.41
117	India	51.93	39.24	45.02	20.74	2434520	1.80
118	Oman	52.51	83.94	31.19	44.67	73370	15.19
119	Trinidad & Tobago	52.98	68.55	33.72	35.67	17760	12.78
120	Kuwait	53.48	95.02	32.86	51.01	89460	21.62
121	Egypt	53.56	49.68	41.99	26.36	246260	2.50
122	Uzbekistan	53.71	52.57	42.97	31.41	112090	3.40
123	Yemen	54.23	48.81	49.56	34.08	9310	0.33
124	Senegal	54.75	39.47	51.08	18.59	9860	0.62
125	Botswana	55.52	44.58	62.10	38.20	8210	3.64
126	Turkmenistan	55.68	71.39	34.41	29.70	71730	12.26

127	Benin	56.03	35.66	61.97	20.42	7910	0.69
128	Nigeria	56.88	35.47	61.96	16.28	130670	0.67
129	South Africa	57.60	47.26	67.41	40.01	433250	7.50
130	Syria	58.86	62.95	47.54	31.86	27910	1.65
131	Gabon	58.92	47.32	57.52	24.83	4610	2.18
132	Iraq	59.21	85.00	36.10	32.34	188140	4.90
133	Iran	59.52	66.85	47.62	33.77	629290	7.69
134	Angola	65.57	51.53	63.52	13.88	27340	0.89
135	Congo - Brazzaville	75.17	81.15	64.27	18.57	3220	0.61

Notes: We report the 2019 ETVI scores as the baseline results to reflect the current status of energy transition vulnerability for each country or region. The ETVI scores for other years are available upon request.

Supplementary note 3: Sensitivity analysis for energy transition vulnerability index

The robustness of the energy transition vulnerability index (ETVI) can be tested by taking uncertainty factors into consideration and conducting a sensitivity analysis. Different settings were tested to identify the composite index's level of sensitivity to the change in parameters – different upper and lower bounds, aggregation methods and a successive exclusion of indicators. The resulting variation of countries' scores and rankings are depicted in **Figure S1**. The countries are ordered according to their median, and the original value of ETVI is marked in red. The results based on alternative upper and lower bounds of 0.01 and 0.99, 0.05 and 0.95 are marked in blue and green, respectively.

a. Sensitivity analysis results for vulnerability rankings

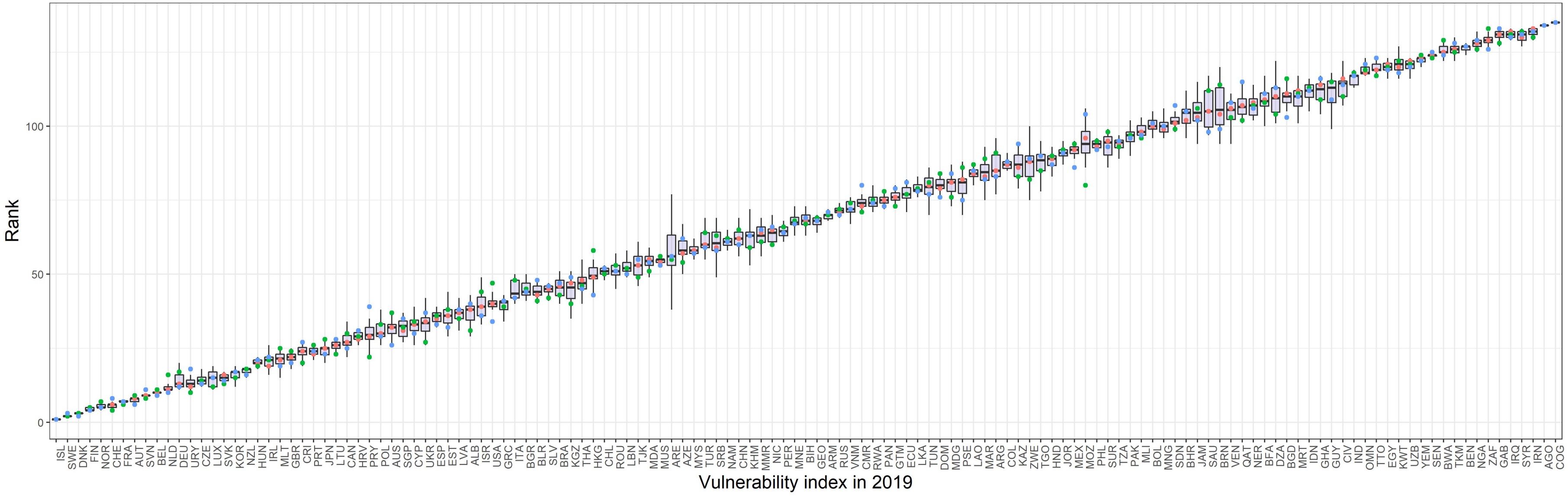

b. Sensitivity analysis results for vulnerability scores

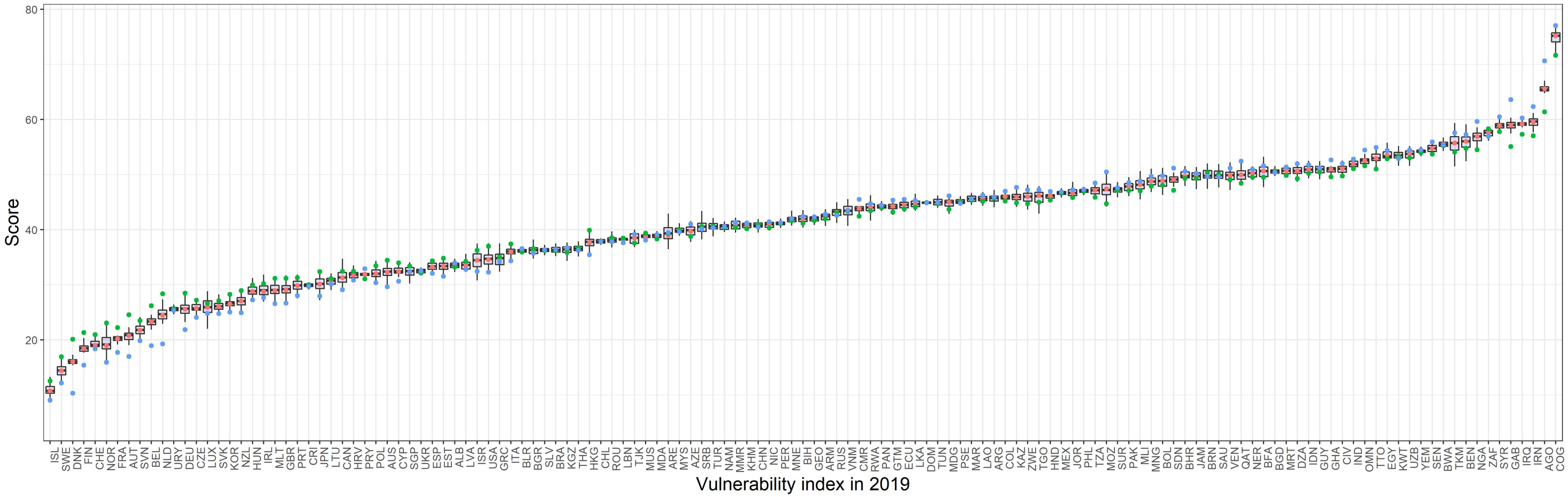

Figure S1. Sensitivity analysis for energy transition vulnerability index

Notes: The robustness of the energy transition vulnerability index (ETVI) is tested by taking different upper and lower bounds, aggregation methods and a successive exclusion of indicators. The countries are ordered according to their median, and the original value of ETVI is marked in red. The results based on alternative upper and lower bounds of 0.01 and 0.99, 0.05 and 0.95 are marked in blue and green, respectively. In each box plot, the central rectangle spans the first quartile Q1 to the third quartile Q3, which is the interquartile range (IQR) (IQR = Q3 to Q1), while the line segment inside the rectangle shows the median. When the maximum observed ETVI scores are greater than Q3 + 1.5 × IQR, the upper whisker is Q3 + 1.5 × IQR. Otherwise, the upper whisker is the maximum observed ETVI score. When the minimum observed ETVI scores are less than Q1 − 1.5 × IQR, the lower whisker is Q1 − 1.5 × IQR. Otherwise, the lower whisker is the minimum observed ETVI score.

The ranking of countries is robust for countries in the top and bottom quintiles of the scale. Although the countries that are listed between those tails reveal a wider interquartile range, the original ETVI ranking remains very close to the median. More than half of the countries only shifted by a maximum of one position from the

median rank. Major differences in scores and ranks are usually witnessed when an indicator that represents a country’s comparative advantage or weakness in transition vulnerability is excluded. However, testing alternative results by excluding indicators showed that both ETVI rankings and scores are not likely to be driven by the outlier in any single dimension in energy transition vulnerability.

Supplementary note 4: Vulnerability index over time

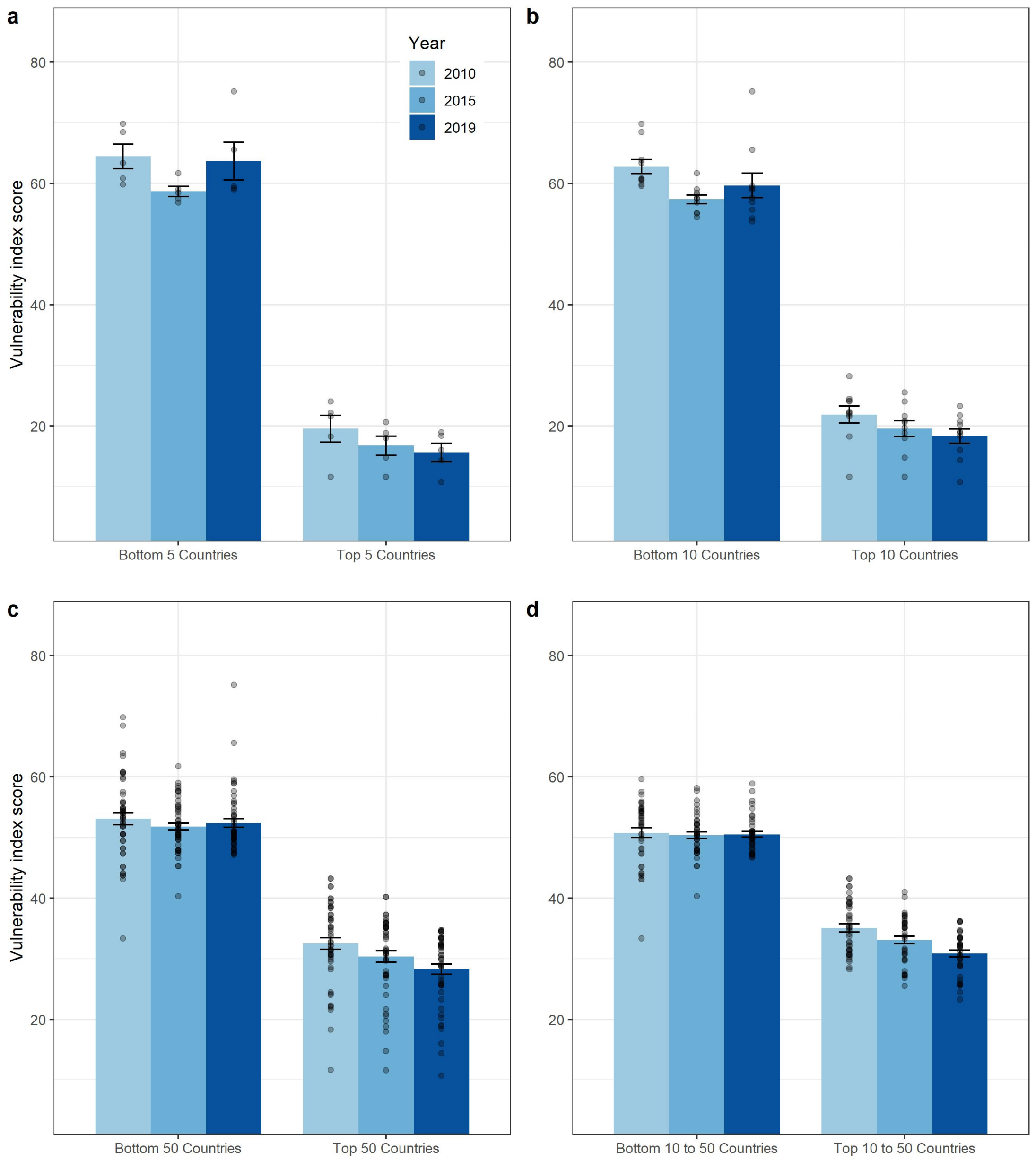

Figure S2. No catching-up effects in transition vulnerability

Notes: a, the top five (best ETVI score) countries and the bottom five (poorest ETVI score) countries in 2010, 2015 and 2019 are compared. **b**, the top ten (best ETVI score) countries and the bottom ten (poorest ETVI score) countries in 2010, 2015 and 2019 are compared. **c**, Top and Bottom 50. **d**, Top and Bottom 10 to 50. The vertical lines within the bar indicate the standard error in ETVI scores.

Supplementary note 5: By party groups for international climate negotiations

We analyze the different energy transition vulnerabilities across seven major party groups in the international climate change negotiations defined by the UNFCCC (**Table S3**). Since each party group shares similarities in climate change, which is the motivation of energy transitions, their energy transition vulnerability may also have common characteristics and dynamics. For example, less developed countries (LDC) have suggested it is unfair that they should bear responsibility for solving a problem they did not cause, while developed nations such as the US have wanted to minimize commitments to avoid burdening their economies. The African Group and the least developed countries group are active in and supportive of all aspects of the climate change negotiating process, with a particular focus on vulnerability and adaptation, both campaigning to prioritize the issue of financial support (UNFCCC, 2022). The EU is well-known as a club of developed countries that strongly advocate for climate ambition, both in international fora and in its bilateral relations with non-EU countries. While the Umbrella countries group also consists of several developed countries (Australia, Canada, Japan, New Zealand, Norway, the United States) plus several former Soviet Union countries (Kazakhstan, the Russian Federation, and Ukraine) are more conservative on climate issues. Therefore, understanding different dynamics in countries' energy transition vulnerability may provide additional information to achieve more inclusive and equitable outcomes in future international climate negotiations.

Table S3. Party groups for international climate negotiations

**African States**	**Arab States**	**European Union**	**Least Developed Countries**	**Like-minded developing countries**	**Small Island Developing States**	**Umbrella Group**
Angola	United Arab Emirates	Austria	Angola	Argentina	Bahrain	Australia
Benin	Bahrain	Belgium	Benin	Bangladesh	Dominican Republic	Canada
Burkina Faso	Algeria	Bulgaria	Burkina Faso	Bolivia	Guyana	Japan
Botswana	Egypt	Cyprus	Bangladesh	China	Jamaica	Kazakhstan
Ivory Coast	Iraq	Czechia	Cambodia	Algeria	Mauritius	New Zealand
Cameroon	Jordan	Germany	Laos	Ecuador	Singapore	Norway
Republic of the Congo	Kuwait	Denmark	Madagascar	Egypt	Suriname	Russia
Algeria	Lebanon	Spain	Mali	Indonesia	Trinidad & Tobago	Ukraine
Egypt	Morocco	Estonia	Myanmar (Burma)	India		United States

Gabon	Mauritania	Finland	Mozambique	Iran
Ghana	Oman	France	Mauritania	Iraq
Morocco	Palestinian Territories	United Kingdom	Niger	Jordan
Madagascar	Qatar	Greece	Rwanda	Kuwait
Mali	Saudi Arabia	Croatia	Sudan	Sri Lanka
Mozambique	Sudan	Hungary	Senegal	Mali
Mauritania	Syria	Ireland	Togo	Malaysia
Mauritius	Tunisia	Italy	Tanzania	Nicaragua
Namibia	Yemen	Lithuania	Yemen	Pakistan
Niger		Luxembourg		Saudi Arabia
Nigeria		Latvia		Sudan
Rwanda		Malta		El Salvador
Sudan		Netherlands		Syria
Senegal		Poland		Venezuela
Togo		Portugal		Vietnam
Tunisia		Romania		
Tanzania		Slovakia		
South Africa		Slovenia		
Zimbabwe		Sweden		

Notes: Seven major party groups in the international climate change negotiations defined by the UNFCCC (UNFCCC, 2022).

Supplementary note 6: Scenario analyses

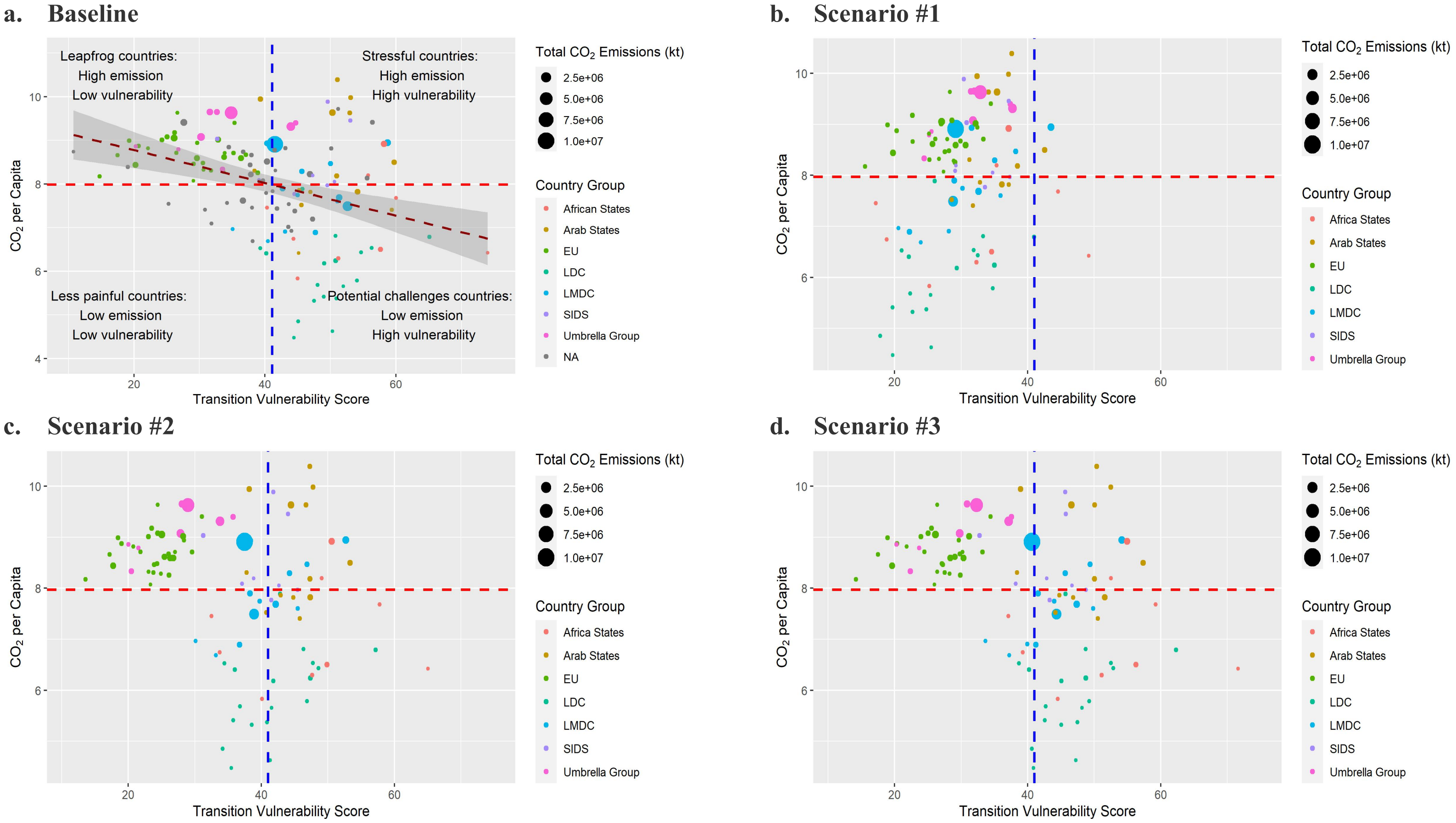

Figure S3. Change in country types in different SDG achievement scenarios

Notes: The relationship between energy transition vulnerability index and CO_2 emission at the climate group and national level in baseline and different SDG achievement scenarios. The vertical and horizontal lines are the mean value in the baseline case for the x and y axis variable so that we set the same criteria for country classification.
